# Sphingolipids in mitochondria—from function to disease

**DOI:** 10.3389/fcell.2023.1302472

**Published:** 2023-11-21

**Authors:** Maryam Jamil, Lauren Ashley Cowart

**Affiliations:** ^1^ Department of Human and Molecular Genetics, Virginia Commonwealth University, Richmond, VA, United States; ^2^ Department of Biochemistry and Molecular Biology, Massey Cancer Center, Virginia Commonwealth University, Richmond, VA, United States; ^3^ Richmond Veteran’s Affairs Medical Center, Richmond, VA, United States

**Keywords:** sphingolipids, mitochondria, ceramide, S1P, NAFLD, cancer

## Abstract

Sphingolipids are not only structural components of cellular membranes but also play vital roles in cell signaling and modulation of cellular processes. Within mitochondria, sphingolipids exert diverse effects on mitochondrial dynamics, energy metabolism, oxidative stress, and cell death pathways. In this review, we summarize literature addressing the crucial role of sphingolipids in mitochondria, highlighting their impact on mitochondrial dynamics, cellular bioenergetics, and important cell processes including apoptosis and mitophagy.

## Introduction

Mitochondria not only constitute the “powerhouse of the cell” but also actively participate broadly in metabolism to maintain cellular homeostasis. Sphingolipids, with their presence in mitochondrial membranes and associations with numerous mitochondrial proteins, have emerged as key regulators of mitochondrial morphology, distribution, and function. Dysregulation of sphingolipid metabolism in mitochondria disrupts numerous mitochondrial processes, leading to mitochondrial fragmentation, impaired bioenergetics, and compromised cellular function. Importantly, such disruptions have been implicated in the pathogenesis of diseases including cancer, cardiovascular disease, Non-alcoholic fatty liver disease (NAFLD), and neurodegenerative diseases, and therefore, how alterations in sphingolipid metabolism may mediate mitochondria-based disease processes is of increasing interest.

Ceramides (Cers) and sphingosine-1-phosphate (S1P) are the two most studied sphingolipid species, and consequently, much of the current knowledge regarding sphingolipids in mitochondria centers around these specific lipid species. As precursors for complex sphingolipids, ceramides serve as the epicenter of the sphingolipid biosynthetic pathway. Alterations in ceramide metabolism contribute to many physiological disorders including fatty liver disease, obesity, cancer, insulin resistance, and cardiovascular disease (CVD) (Holland and Summers, 2008; Boini et al., 2010; Summers, 2010; Furuya et al., 2011; Nikolova-Karakashian, 2018; Alessenko et al., 2019). Additionally, S1P, arising from ceramide catabolism, functions both intracellularly and extracellularly in additional regulatory roles. Both ceramide and S1P have been shown to regulate mitochondrial function influencing mitochondrial dynamics, bioenergetics, and cell death pathways. In this review we have presented a brief overview of the sphingolipid species and their metabolism followed by a detailed summary of the role of ceramides and S1P in mitochondrial function.

## Sphingolipid synthesis/metabolism: A brief overview

**FIGURE 1:**
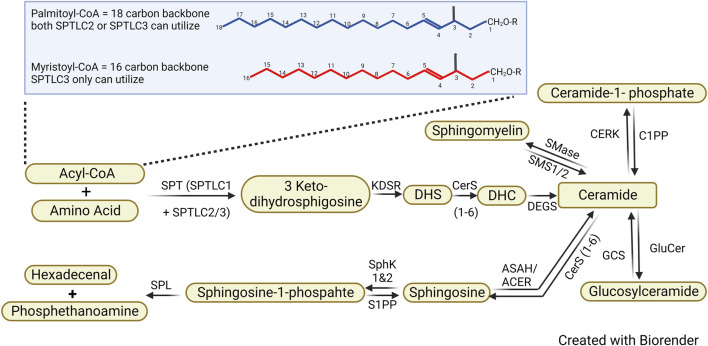
Sphingolipid *de novo* synthesis pathway. Sphingolipid synthesis starts with the condensation of an amino group with Acyl-CoA, leading to the formation of 3 Keto-dihydrosphingosine which is ultimately converted into dihydroceramide (DHS). Dihydroceramide is subsequently desaturated to ceramide, a central molecule in sphingolipid metabolism. Ceramide serves as a precursor for various sphingolipids, including sphingomyelin, Ceramide-1-phosphate, glucosylceramides, and sphingosine, through the addition of different headgroups.

*De novo* synthesis of sphingolipids starts in the endoplasmic reticulum (ER) where serine palmitoyltransferase (SPT) catalyzes the first committed step in sphingolipid biosynthesis, the condensation of an amino acid with acyl-CoA into 3-Ketodihydrophingosine (KDHSph) (Figure 1). The second step requires the NADPH-dependent enzyme 3-Ketodihydrosphingosine reductase, which rapidly converts KDHSph to dihydrosphingosine (DHS). DHS, containing one fatty chain, is acylated to form two-chained dihydroceramides (DHC) by the enzyme (dihydro)ceramide synthase (CerS). DHC is converted into ceramide by the dihydroceramide desaturase (DES). All of these enzymes are ER-resident, and therefore, *de nove* ceramide production occurs largely in the ER. After their *de novo* synthesis, ceramides are shuttled to the Golgi apparatus via vesicular transport or ceramide transport protein (CERT), where they serve as substrates for production of the complex sphingolipids sphingomyelin and glycosphingolipids. This occurs via three major enzymes: ceramide galactosyltransferase, glucosylceramide synthase, and sphingomyelin synthase yielding galactosylsphingolipids, glucosylceramides, and sphingomyelin, respectively. In addition to serving as the precursor of complex sphingolipids, ceramide can also be phosphorylated by ceramide kinases (CERK) to produce ceramide-1-phosphate (C1P), a bioactive lipid that activates phospholipase A2, serving as a critical node of cross-talk between sphingolipid metabolism and eicosanoid production (Lamour and Chalfant, 2005). Ceramides also undergo catabolism through de-acylation to make sphingosine, which can be re-acylated to remodel ceramide N-acyl chain lengths through the sphingosine salvage pathway or phosphorylated by sphingosine kinases (SphK1 and SphK2) to make sphingosine-1-phosphate (S1P). Numerous forward and reverse enzymatic steps work to shift sphingolipid metabolites, once synthesized, through different branches of the pathway. Importantly, however, the only way sphingolipids can exit the metabolic pathway is by S1P lyase which catabolizes S1P into the non-sphingolipid molecules, fatty aldehyde and ethanolamine phosphate. Therefore, inhibiting S1P degradation can lead to increases of cell sphingolipids.

The rate-limiting step in sphingolipid synthesis occurs via a multimeric enzyme SPT, which consists of catalytic subunits, SPTLC1, SPTLC2, and/or SPTLC3, and several regulatory subunits including ssSPTa/b and the ORMDL proteins. The complex occurs in various configurations (SPTLC2 vs. 3, inclusion of ssSPTa or b, ORMDL) which convey distinct catalytic properties to generate a multitude of structurally variable sphingoid bases. In the canonical complex, SPTLC1 heterodimerizes with SPTLC2 to condense 2-carbon serine with 16-carbon palmitoyl-CoA giving rise to canonical sphingolipids with an 18-carbon sphingoid backbone (d18:0 DHS) (Nagiec et al., 1994). Alternatively, the SPT heterodimer comprising SPTLC1-SPTLC3 can also utilize 14-carbon myristoyl-CoA to generate atypical sphingolipids with a 16-carbon sphingoid backbone (Hornemann et al., 2006). Under the influence of SPT small subunits ssSPTa and ssSPTb, the SPT complex can also utilize stearoyl-CoA to generate d20:0 DHS (Han et al., 2009; Harmon et al., 2013). Interestingly, point mutations in SPT subunits are associated with disease and have been demonstrated to alter the amino acid selectivity of the enzyme generating deoxy- or desoxysphingolipids derived from SPT utilization of alanine or glycine, respectively (Eichler et al., 2009). Roles for these more recently described bases are under investigation, and disease pathology may not only arise from lack of canonical sphingolipids, but also from the deoxy- and desoxysphingolipids, which cannot be metabolized to downstream lipids and also may have their own toxic properties (Kramer et al., 2015). Importantly, the SPT complex is subject to inhibition in the presence of high intracellular ceramides, which bind and activate the ORMDL proteins, thereby decreasing SPT activity (Davis et al., 2018).

In addition to variations in the sphingoid backbone, ceramides (and, consequently, any downstream complex sphingolipid containing ceramide), also vary by the attached acyl group at the C2-amino group, which is determined by the specific CerS isoform catalyzing their production. Since the cloning of mammalian CerS isoforms, this area of sphingolipid research has ballooned as data support distinct tissue expression, regulation in disease, and inverse/compensatory changes in CerS enzyme isoforms upon experimental manipulation. As understanding of the specific roles of these diverse ceramide species increases, developing isoform-specific inhibitors has arisen as an urgent need.

## Biological functions of sphingolipid species

### Ceramides

Ceramides act as key mediators in signaling pathways involved in several cellular functions including apoptosis, cell growth, and differentiation (Spiegel and Merrill, 1996; Bartke and Hannun, 2009; Young et al., 2013). As outlined above, ceramides can be generated through *de novo* synthesis (SPT to CerS), acylation of sphingosine by ceramidase, or catabolism of complex sphingolipids such as sphingomyelin (SM) via SMS, galactosylceramides via galactosylceramidase, and glucosylceramides via glucosylceramidase (Hannun and Obeid, 2018). Previously, ceramides were considered one single class of sphingolipids but, as noted above, it has become clear that Ceramide synthase isoforms (CerS1-6) have distinct substrate preferences for the incorporation of fatty acids with different chain lengths thereby generating an array of distinct species (Venkataraman et al., 2002; Riebeling et al., 2003; Laviad et al., 2008). In mammals the length of the acyl-chains varies greatly from medium chain fatty acids 12–14C, long chain fatty acids 16–20C, very long chain fatty acids 22–26C, and ultra-long chain fatty acids >26C, and these distinct species have different functions (Grosch et al., 2012). These differences amongst ceramides with different acyl chain lengths play important roles in maintaining mitochondrial homeostasis (Di Paola et al., 2000; Raichur et al., 2014).

### Sphingomyelin

Ceramides are converted into many complex sphingolipids including sphingomyelin (SM). Sphingomyelin synthase (SMS); a Golgi bound enzyme; catalyzes the transfer of phosphocholine moiety from phosphatidylcholine (PC) to ceramides producing SM and diacylglycerol (DAG). DAG is an important regulator of cell growth and has been shown to compete against ceramide induced apoptosis (Obeid et al., 1993). Therefore, it has been proposed that SMS activity is critical for cell growth and survival as it regulates the balance between the pro-apoptotic mediator ceramide and pro-survival mediator DAG. Several lines of evidence support this hypothesis where stimulation of SMS enhanced DAG binding activity whereas downregulation of SMS inhibited DAG localization to Golgi (Villani et al., 2008). Additional data suggest a specific role for SM irrespective of DAG. For example, a mouse lymphoid cell line with defective SMS activity could not grow in culture whereas exogenous treatment with SM restored cell growth (Yamaoka et al., 2004). Moreover, SPT mutant hamster ovarian cells died in the absence of exogenous sphingolipids but were rescued with only with SM treatment but not by treatment with other complex sphingolipids (Merrill, 1983). Sphingomyelin is hydrolyzed by either a lysosomal enzyme acidic sphingomyelinase (aSMase) or plasma membrane neutral sphingomyelinase (nSMase), which removes the phosphocholine headgroup releasing ceramides. Defects in SMase activity lead to accumulation of SMs in the lysosomes resulting in a lysosomal storage disorder called Niemann Pick Disorder (NPD), a neurodegenerative disease resulting in premature death (Brady et al., 1966). NPD and numerous other lysosomal storage diseases arising from mutations in sphingolipid catabolic enzymes demonstrate that a balance between sphingolipid synthesis and degradation is essential for normal cellular function.

### Glycosphingolipids

The biosynthesis of glycosphingolipids (GSLs) starts with an addition of sugar groups to ceramides, and they are characterized based on the sugar group added. Addition of β-linked galactose or glucose gives rise to galactosylceramide (GalCer) and glucosylceramides (GlcCer), respectively. The synthesis of GlcCer happens in the cytoplasmic face of the ER and early golgi from where it translocates into the lumen of the golgi, whereas GalCer is synthesized in the lumen of the ER and is trafficked through the golgi. Glucosylceramide synthase (GLS), a transmembrane golgi protein, utilizes UDP-glucose and ceramides to synthesize GlcCer, which is a major precursor of hundreds of ganglioside and globoside GSLs. GlcCers are essential in mammalian development and are likely involved in cell-cell recognition and differentiation (Yamashita et al., 1999). Alternatively, Galactosylceramide transferase (GST) utilizes UDP-galactose to transfer the galactose group to ceramide resulting in GalCer. Galactosylceramides are enriched in schwann cells, oligodentrocytes, myelin where they play crucial roles in brain development (Zoller et al., 2005). GSLs are catabolized in endosomes and lysosomes where lysosomal glycosidases cleave off the sugar residues from the non-reducing end and the remaining fatty acids and sphingoid bases are either degraded or recycled through the salvage pathway. Defects in any of the lysosomal glycosidase protein activity can result in multiple lysosomal storage disorders including Gaucher, Sandhoff, Tay Sachs, Fabry disease and gangliosidosis, each of these disorders is associated with an accumulation of one or more unique GSL (Platt, 2014). Little is known about potential functions of GSL with respect to mitochondria, though mass spectrometric analysis of mitochondrial sphingolipid composition in murine livers has identified the presence of ceramides, sphingomyelins, and gangliosides (Bird et al., 2013). Emerging evidence suggests that complex glycosphingolipids regulate inter-organellar contact sites including ER-mitochondria and potentially mitochondria-lysosome membrane contact sites, though generally, little is known about mitochondrial roles of complex sphingolipids (Weesner et al., 2023).

### Sphingosine-1-phosphate

Sphingosine produced by de-acylation of ceramides can be phosphorylated by sphingosine kinases (SKs) to produce Sphingosine-1-phosphate (S1P) (Gandy and Obeid, 2013). Ceramidases (CDases) are pivotal players in this conversion, as they facilitate the transition from ceramide to sphingosine by cleaving the attached fatty acid group (Mao and Obeid, 2008). Presently, five distinct ceramidase genes have been identified, categorized based on the optimal pH conditions required for their activity. Sphingosine kinase 1 and 2 (SK1/2), members of the DAG kinase family, utilize ATP to phosphorylate the C-1 hydroxy group of sphingosine or dihydrosphingosine resulting in the production of S1P or dihydro-S1P (dhS1P) respectively (Gandy and Obeid, 2013; Orr Gandy and Obeid, 2013). The two isoforms of SK, SK1 and SK2, have different subcellular localizations and can be regulated by various signaling pathways. SK1 is mainly located in the cytosol, while SK2 is primarily found in the nucleus, endoplasmic reticulum, and mitochondria (Igarashi et al., 2003; Neubauer and Pitson, 2013; Hatoum et al., 2017). S1P acts as an intercellular, intracellular, and extracellular mediator in many cellular signaling pathways. It is critical for many physiological and pathophysiological processes including cancer, inflammation, diabetes, obesity, and cardiomyopathies (Hla, 2004; Jin et al., 2016; Nagahashi et al., 2018; Obinata and Hla, 2019; Jozefczuk et al., 2020; Green et al., 2021; Liu et al., 2022). For extracellular functions S1P can be transported across the plasma membrane through ATP binding cassette family members (ABC) or spinster two (spns2) transporters (Kobayashi et al., 2009; Sato et al., 2007; Spiegel et al., 2019). S1P can then bind a receptor on the same cell (autocrine), a nearby cell (paracrine), or enter the circulation to activate distal receptors (autocrine). At least five different G protein-coupled receptors for S1P are known (S1PR1-5) (Sanchez and Hla, 2004). These receptors play important roles in mediating S1P signaling and can activate various intracellular signaling pathways (Tsai and Han, 2016). S1P can also be dephosphorylated into its precursor Sph via different lipid phosphatases or S1P specific phosphatases (Mandala et al., 2000; Johnson et al., 2003; Ogawa et al., 2003). S1P can be broken down by S1P lyase (S1PL) into fatty aldehyde and phosphoethanolamine, and this is the only irreversible reaction in the sphingolipid metabolic pathway through which a sphingoid base is broken down into non-sphingolipid components (Bourquin et al., 2010; Serra and Saba, 2010).

## Sphingolipids in mitochondria

### Ceramides and mitochondria

**FIGURE 2:**
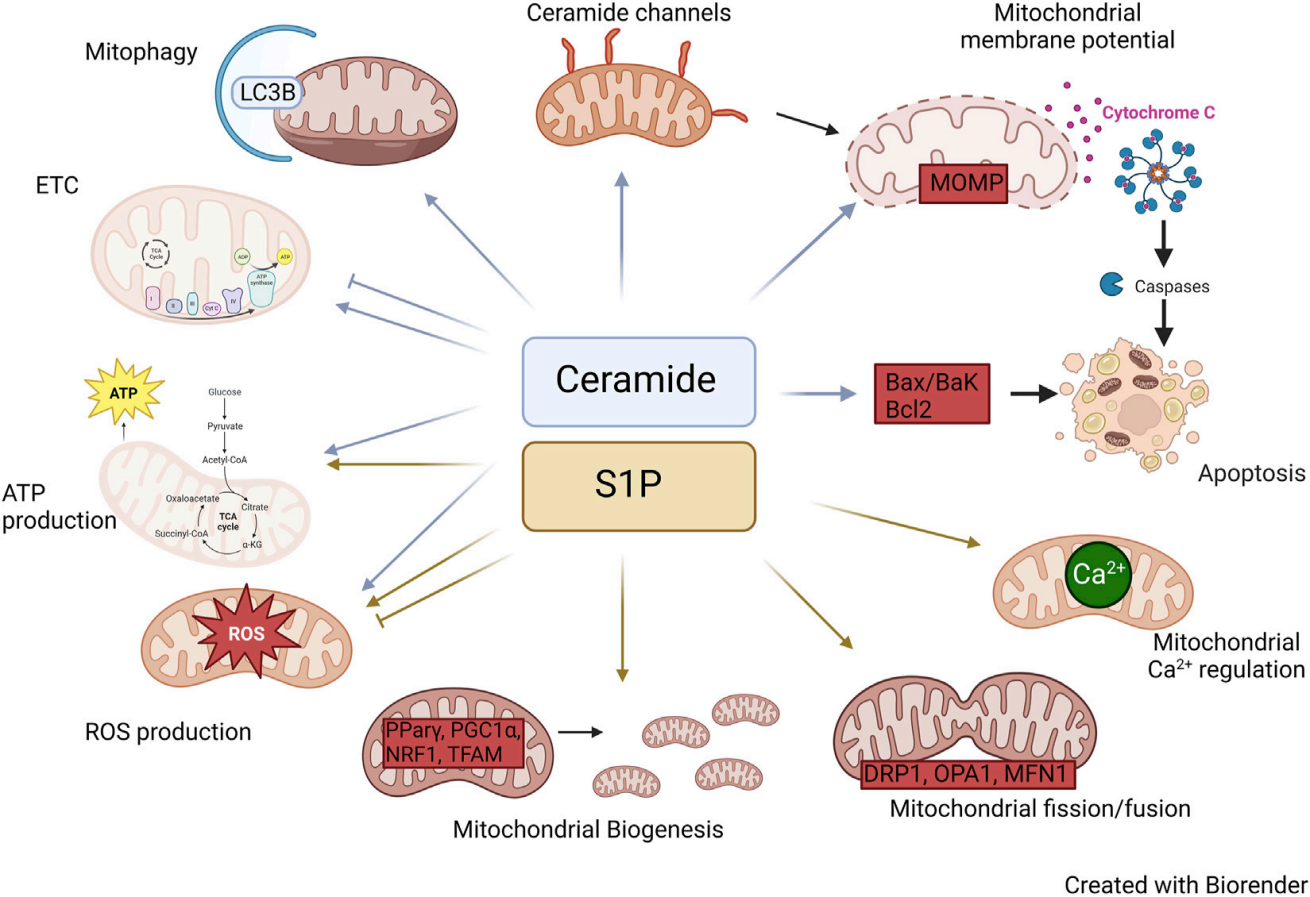
Roles of Ceramide and S1P in mitochondria. Diagram illustrating the mechanisms by which ceramide and S1P alter mitochondrial biology.

Ceramides have been the focus of significant research with respect to their role in the mitochondria. Ceramides, largely synthesized in the ER, can be transferred to the mitochondria via the mitochondrial associated membranes (MAMs), which serve as a bridge between the ER and mitochondria (Stiban et al., 2008). While ceramides are mainly produced at the cytosolic surface of the endoplasmic reticulum (ER), recent reports have shown the presence of CerS, SMase, and reverse ceramidase activity in mitochondria, suggesting localized synthesis of ceramides can also occur within the organelle (Shimeno et al., 1998; El Bawab et al., 2000; Birbes et al., 2001; Novgorodov et al., 2011). However, this pathway in less common compared to the transfer of ceramides from the ER. The collective operation of these pathways plays a pivotal role in regulating the ceramide levels within the mitochondria (Figure 2; Table 1).

**TABLE 1:** Role of ceramides and S1P in mitochondrial pathways.

SL species	Pathway	Sphingolipid Level(s)
Ceramides	ETC	• ↓ CI activity with C2-ceramide Gudz et al. (1997) • ↑ CIV activity with C2-ceramide Di Paola et al. (2000) • ↓ CIV activity with C16-ceramide Di Paola et al. (2000); Zigdon et al. (2013) • ↓ CIV activity in CerS2-deficient mice Zigdon et al. (2013)
	ROS production	• ↑ ROS production with C16-ceramide Di Paola et al., (2000); Zigdon et al. (2013) • ↓ ROS production with C2 and C6-ceramides Gudz et al. (1997); Di Paola et al. (2000)
	MOMP	• ↓ MOMP with C8-ceramide Arora et al. (1997) • ↑ Mitochondrial membrane permeability transition with C2, C6, C8-ceramides Arora et al. (1997) • ↓ MOMP by ceramide induced formation of pores within the mitochondrial membrane Siskind and Colombini. (2000) • ↓ MOMP by internalization of ceramide platforms in plasma membrane and the exchange of ceramides between plasma membrane and mitochondria Babiychuk et al. (2011)
	ATP production	• ↓ ATP production with C8-ceramide Arora et al. (1997)
	Mitophagy	• ↑ Mitophagy with CerS1 derived C18-ceramide Jiang and Ogretmen. (2013) • CerS6 derived C16-ceramide interacts with mitochondrial fission factor Hammerschmidt et al. (2019) • C18 ceramide interaction with LC3B Sentelle et al. (2012) • ↑ Mitophagy involving the recruitment of LC3B-containing autophagosomes by mitochondrial ceramide Sentelle et al. (2012); Jiang and Ogretmen. (2013)
	Mitochondrial dynamics	• CerS6 derived C16-ceramide interacts with mitochondrial fission factor Hammerschmidt et al. (2019) • Ceramides can interact with VDAC2 Dadsena et al. (2019)
S1P	Mitochondrial Biogenesis	• Direct regulation via PPARγ, PGC1α Moon et al. (2012); Chen et al. (2016); Liu et al. (2016); Luo et al. (2016); Weske et al. (2018); Meyer Zu Reckendorf et al. (2020) • Indirect regulation via TFAM, NRF1, ERK, MAPK, AMPK, p38 Stechschulte et al. (2014); Banks et al. (2015); Dennhardt et al. (2019) • ↑ Mitochondrial biogenesis with S1P via S1PR2 Shen et al. (2014)
	ATP Production	• ↑ ATP production with S1P via S1PR2 Shen et al. (2014)
	ROS production	• ↑ ROS production with activation of S1P signaling Yu et al. (2018); Botta et al. (2019); Liu and Tie. (2019); Botta et al. (2020) • ↓ ROS production with inhibition of S1P signaling Pyszko and Strosznajder. (2014); Sivasubramanian et al. (2015); Oancea-Castillo et al. (2017) • ↑ ROS production with activation of S1P signaling Golan et al. (2012); Kim et al. (2014); Lin et al. (2016); Ha et al. (2020); Li et al. (2020)
	Ca^2+^ Homeostasis	• ↑ Mitochondrial Ca^2+^ with overexpression of SK1 Pulli et al. (2019) • ↓ Mitochondrial Ca^2+^ with exogenous S1P Agudo-Lopez et al. (2010)
	Mitochondrial dynamics	• ↑ DRP1 with exogenous S1P via S1PR3 Brand et al. (2018) • ↓ DRP1 with inhibition of S1P via S1PR2 Chen et al. (2019) • ↓ OPA1 and MFN1 with SK inhibitor Hong et al. (2018) • Altering the expression of S1PR1 perturbed the equilibrium of gene expression related to mitochondrial fission and fusion Bajwa et al. (2015)

The presence or absence of specific ceramides directly influence the workings of the electron transfer chain (ETC.). The ETC consists of four multimeric protein complexes (CI-IV), two electron carriers (ubiquinol and cytochrome c). Numerous studies have provided evidence demonstrating the involvement of ceramides in the regulation of ETC complex activity. For example, in isolated mitochondria, exogenous addition of C2 ceramide inhibited CI activity (Gudz et al., 1997). Moreover, investigations conducted on heart mitochondria have demonstrated that ceramides have inhibitory effects on both CI and CIV activities. Interestingly, the effects of long-chain and short-chain ceramides on CIV catalytic activity were found to be contrasting. Specifically, C2 ceramides were shown to stimulate CIV activity, whereas C16 ceramides exhibited inhibitory effects (Di Paola et al., 2000). These findings indicate that both the quantity and quality of ceramides can influence the activity of the ETC but the exact mechanism of how ceramides effect ETC complexes is not yet fully understood. However, multiple *in vivo* studies also support roles for CerS and sphingolipids in ETC complex function. For example, CerS2-deficient mice showed decreased CIV activity caused by drastic changes in liver mitochondrial lipid composition that also resulted in accumulation of C16:0-ceramide, sphingosine, and dihydrosphingosine (Zigdon et al., 2013). Exogenous treatment with C16 ceramide, sphingosine, or dihydrosphingosine reduced CIV activity, and together, they showed an additive effect.

Mitochondria are the main source of intracellular reactive oxygen species (ROS) production, and electrons leaking through the ETC react with oxygen species to generate ROS. Furthermore, defects in ETC complexes often increase ROS generation. Exogenous treatment of isolated mitochondria with ceramides as well as murine models with mitochondrial ceramide accumulation have shown an increase in ROS production (Gudz et al., 1997; Di Paola et al., 2000; Zigdon et al., 2013). The exact mechanism by which ceramides alter ETC activity is still not completely understood, but we hypothesize that ceramides may induce alterations in the lipid composition of the mitochondrial membrane and/or affect the formation of ETC assembly.

Ceramides have been demonstrated to reduce mitochondrial membrane potential. Arora et al. (1997) have shown that cultured hepatocytes treated with C8-ceramide had a decrease in mitochondrial membrane potential and ATP depletion. Interestingly, a similar effect was not observed in cultured hepatocytes when treated with dihydro-C8-ceramide. Furthermore, treatment of isolated mitochondria with ceramides induced mitochondrial membrane permeability transition (MMPT) which was dependent on the time, concentration, as well as the length of acyl chain as C8-ceramide was the most potent inducer compared to C2 and C6-ceramides (Arora et al., 1997). These investigations have greatly elucidated the potential for distinct functions of distinct ceramide species in mitochondrial biology. Nevertheless, there are several limitations associated with the use of exogenous ceramides. For instance, exogenous ceramides may not precisely mirror the specific ceramide subspecies found in a particular cellular context, potentially resulting in diverse outcomes. However, distinct roles for specific N-acyl chain length ceramide species gains support from studies, for example within cells ceramides like C16 and C22 form channels which are inhibited by incorporation of other species of ceramide in the channel (Di Paola et al., 2000; Stiban and Perera, 2015). It is important to note that, while exogenous ceramides are remodeled to reflect endogenous species by the sphingosine salvage pathway (i.e. ceramidase to generate sphingosine, followed by re-acylation of sphingosine by endogenous CerS isoforms), exogenous addition may lead to alterations in intracellular localization, and, moreover, may cause adverse effects in the plasma membrane.

### Apoptosis

Apoptosis is a programmed cell death process that occurs in multicellular organisms to remove damaged or unnecessary cells. There are two major apoptotic pathways: Extrinsic and intrinsic. The extrinsic pathway is activated by extracellular signals such as Fas ligand and tumor necrosis factor (TNF), while the intrinsic pathway is initiated by intracellular stress signals such as DNA damage and oxidative stress (Elmore, 2007). Mitochondria play a crucial role in the intrinsic apoptotic pathway by responding to intracellular stress signals and initiating mitochondrial outer membrane permeabilization (MOMP), which leads to the release of pro-apoptotic factors and the activation of caspases, ultimately resulting in apoptosis (Elmore, 2007).

The role of ceramides in apoptosis has been extensively studied. The study conducted by Obeid et al. (1993) was a pioneering paper demonstrating ceramides induced apoptosis. Importantly, they demonstrated that ceramide could induce apoptosis not only when added exogenously to cells but also when endogenous levels of ceramide were manipulated after treatment with TNFα, which resulted in sphingomyelin hydrolysis. This finding placed ceramide as an endogenous mediator of apoptosis (Obeid et al., 1993). Numerous studies conducted since then have demonstrated elevated ceramide levels in multiple cell types, and many studies have demonstrated increased mitochondrial ceramide levels following diverse pro-apoptotic stimuli.

Ceramides are highly insoluble and therefore reside largely within cell membranes. Babiychuk et al. (2011) showed that under apoptotic conditions ceramide platforms formed within the plasma membrane were internalized, coming in contact with the mitochondrial membrane, which suggests potential ceramide exchange between the plasma membrane and the mitochondria. This phenomenon was termed the “kiss of death” as it led to the permeabilization of the mitochondrial outer membrane ultimately resulting in apoptosis (Babiychuk et al., 2011). Ceramides can also form large stable pores within the mitochondrial membrane, thereby perturbing the mitochondrial membrane potential. This pore-forming capacity is exhibited by both long and short-chain ceramides, with the size of the pore contingent upon the concentration of ceramides present (Siskind and Colombini, 2000). One of the earliest events triggered by increased ceramide levels is the release of proteins from the mitochondrial intermembrane space (Hea et al., 2002; Lin et al., 2004). This activates caspases, the proteases that play a crucial role in apoptosis. Moreover, ceramide-induced apoptosis is also associated with excessive mitochondrial ROS generation, a hallmark of mitochondrial dysfunction. As mitochondrial dysfunction progresses, there is a collapse in MOMP, which further exacerbates the production of ROS and activates the intrinsic apoptotic pathway (Bock and Tait, 2020). Ultimately, these events lead to the activation of caspase 3 and the cleavage of various substrates that initiate the dismantling of the cell. MOMP is a critical step in determining cell fate and certain studies indicate that MOMP cannot be induced by ceramide alone and requires the synergistic action of Bax. Bax utilizes ceramide pores to integrate into the outer membrane of the mitochondria, thereby inducing loss of MOMP (Ganesan et al., 2010; von Haefen et al., 2002; Lee et al., 2011; Martinez-Abundis et al., 2009). Furthermore, various interventions that suppress mitochondrial dysfunction have been shown to suppress ceramide-induced apoptosis, including cyclosporine A, Bcl-2, and Bcl-xL (Karasavvas et al., 1996; Zhang et al., 1996; Wiesner et al., 1997; Pacher and Hajnoczky, 2001). These interventions can block or attenuate the early events induced by ceramide, such as the release of proteins from the mitochondrial intermembrane space and the generation of ROS, and thus prevent or delay the progression of the apoptotic pathway. Moreover, several studies have demonstrated that the use of pharmacological agents (myriocin) or siRNA targeted towards sphingolipid synthesis can impede cell death (Mullen and Obeid, 2012). In contrast, ceramide metabolism has been found to protect cells from apoptotic stimuli and from ceramide-induced apoptosis (Liu et al., 2008). This suggests mitochondrial presence of ceramides is vital for normal maintenance of mitochondrial function, and upon dysregulation, ceramides can become pro-apoptotic.

Ceramides not only play a crucial role in determining cell fate by inducing apoptosis but also regulate mitochondrial fate by controlling mitophagy (Dany and Ogretmen). Mitophagy can play a significant role in both cell death and cell survival by degrading mitochondria before initiation of the apoptotic cascade or by degrading damaged mitochondria to prevent further injury. Studies have revealed that CerS1 and its product C18-ceramide can induce mitophagy independent of Bak, Bax, or caspases (Sentelle et al., 2012). Sentelle et al. demonstrated that ceramide directly binds to lipidated LC3B. This binding was confirmed through experiments in which cells expressing CerS1 were labeled with biotin-sphingosine, resulting in the generation of biotin-C18-ceramide upon CerS1 induction. Pull-down assays confirmed the direct interaction between LC3B and C18 ceramide (Sentelle et al., 2012). This study also shows evidence indicating that C18-ceramide has a direct interaction with lipidated LC3 on the mitochondrial membrane, triggering the process of mitophagy. Ceramides have a higher affinity for the PE-conjugated LC3-II than LC3-I, which involves the central hydrophobic domain of LC3, likely due to structural similarities between CERT and this domain. Moreover, ceramide acts as a novel receptor at the mitochondrial membranes to recruit LC3-II-containing autophagosomes to the mitochondria (Sentelle et al., 2012; Jiang and Ogretmen, 2013). Similarly, C16-ceramide also exhibited similar results but only when localized to the mitochondria. This suggests that the subcellular location of ceramide, and the length of the fatty acid chain, plays a significant role in regulating ceramide-induced mitophagy (Sentelle et al., 2012). This concept gains additional support from studies using mitochondria-directed ceramide analogs promoted mitophagy in a manner dependent on N-acyl chain length, with the longest lengths promoting the most intense mitophagic response (Law et al., 2018). Recent findings have revealed that ceramides can directly interact with the voltage-dependent anion channel (VDAC2), which serves as both a regulator of mitochondrial metabolic and ion flow and a gatekeeper, managing the equilibrium between mitochondrial and cytosolic Bax pools. Therefore, ceramides can also induce cell death through their interaction with VDAC2 (Dadsena et al., 2019).

Overall, the regulation of ceramide-induced apoptosis and mitophagy represents a potential therapeutic approach for various diseases. For example, CerS6 derived C16 sphingolipid has been shown to interact with mitochondrial fission factor (Mff), regulating mitochondrial dynamics and insulin resistance in obesity (Hammerschmidt et al., 2019). Further research is needed to better understand the complex roles of ceramides in mitochondria (Figure 2); moreover, thorough understanding of the relationships between CerS isoforms and distinct molecular species of ceramide will be essential for developing safe and effective ceramide-based therapeutics.

### S1P and mitochondria

S1P, or sphingosine-1-phosphate, plays important roles in various physiological processes, including cell proliferation, survival, and differentiation. The regulation of S1P levels in the cell is critical for optimal cellular function and can be achieved through the regulation of its synthesis (SK1/2), degradation (SPL, S1P phosphatases), and transport (S1PRs, SPNS2, and ABC). S1P has been extensively studied for its roles in mitochondrial survival, biogenesis, dynamics, and respiration, making it, alongside ceramides, one of the most studied sphingolipids in relation to mitochondria.

The process of mitochondrial biogenesis requires replicating mitochondrial DNA, synthesizing mitochondrial proteins, and assembling the mitochondrial membrane. Research indicates that S1P can regulate PPARγ (Peroxisome Proliferator Activated Receptor Gamma), a nuclear receptor, which, with its binding partner PGC1α (Peroxisome proliferator-activated receptor gamma coactivator 1-alpha), controls mitochondrial biogenesis. In various cell types as well as murine models, knockdown of S1P has been shown to either increase or decrease PPARγ expression, while S1P can also serve as a ligand to directly boost PPARγ expression levels (Moon et al., 2012; Chen et al., 2016; Liu et al., 2016; Luo et al., 2016; Weske et al., 2018; Meyer Zu Reckendorf et al., 2020). Furthermore, S1P can also regulate PGC1α, a coactivator of PPARγ. This complex activates nuclear respiratory factor 1 (NRF1) and mitochondrial transcription factor A (TFAM) both of which regulate mitochondrial DNA replication and transcription. Additionally, S1P can modulate the expression of transcription factors such as ERK, MAPK, AMPK, and p38, which can in turn impact PPARγ expression, highlighting the potential for direct and indirect regulation of PPARγ by S1P (Stechschulte et al., 2014; Banks et al., 2015; Dennhardt et al., 2019). Receptor-mediated regulation of mitochondrial biogenesis gains support from several studies including one HepG2 cells, in which exogenous S1P through S1PR2 promoted mitochondrial biogenesis via upregulation of ATP synthesis, mitochondrial DNA replication and transcription through PGC1α and its downstream targets (Shen et al., 2014). Additionally, inhibitor of S1PRs FTY720 has shown to inhibit PPARγ transcription (Moon et al., 2012).

Several studies have examined the involvement of S1P in oxidative stress. Activation of S1P signaling through S1P receptors has been found to safeguard cardiomyocytes against high-fat diet or palmitate-induced lipotoxicity, which is accompanied by a decrease in ROS, inflammation, and overall cell proliferation (Botta et al., 2019). Similar findings have been reported in both normal and cancerous cells (Yu et al., 2018; Liu and Tie, 2019; Botta et al., 2020). Furthermore, downregulation of S1P regulators increased ROS production in multiple cell types including glioblastoma cells, neuroblastoma cells as well as various tumor cells (Pyszko and Strosznajder, 2014; Sivasubramanian et al., 2015; Oancea-Castillo et al., 2017). For example, impaired S1P regulation in lung endothelium increased hyperoxia-mediated ROS generation and lung injury (Harijith et al., 2016). On the contrary, enhanced S1P signaling in hepatocytes, epithelial cells, hematopoeitic cells, and breast carcinoma cells have shown an increase in overall ROS production (Golan et al., 2012; Kim et al., 2014; Lin et al., 2016; Ha et al., 2020; Li et al., 2020). This shows that depending on the cell type S1P can have different effects.

S1P also plays a role in mitochondrial Ca^2+^. Homeostasis. Calcium is an important intracellular messenger that regulates metabolism, gene expression, cell cycle, and many other cell processes. Mitochondria take up calcium from the cytoplasm which is mediated by the mitochondrial Ca^2+^ uniporter protein complex (MCU). MCU can sense changes in cytoplasmic calcium levels and adjust the rate of calcium uptake accordingly. Once inside the mitochondria, calcium regulates various mitochondrial functions, including ATP production, metabolism, and reactive oxygen species (ROS) production. However, high levels of calcium can induce the opening of the mitochondrial permeability transition pore (MPTP), causing the collapse of the mitochondrial membrane potential and the release of pro-apoptotic factors. Therefore, the regulation of mitochondrial calcium levels is critical for maintaining normal mitochondrial function and preventing cell death. Supporting a role for S1P in this process, overexpression of SK1 in HeLa cells increased mitochondrial Ca^2+^ (Pulli et al., 2019). On the other hand, exogenous S1P in neuroblastoma cells attenuated mitochondrial Ca^2+^ accumulation during ischemic conditions (Agudo-Lopez et al., 2010). These findings collectively indicate that the impact of S1P on mitochondrial calcium levels may vary depending on the cell type, signaling mechanism (e.g. intracellular or receptor-mediated), and/or other experimental variables.

Mitochondrial function is also influenced by the dynamics of fission and fusion, where a balance is crucial. Healthy mitochondria undergo fusion, while damaged ones undergo fission to eliminate defective components. Various proteins, such as DRP1 for fission and OPA1, mitofusin 1 and 2 (MFN1/2) for fusion, mediate this balance (Rana et al., 2017). A study by Brand et al. (2018) demonstrated that exogenous S1P treatment via S1PR3 in cardiomyocytes increased DRP1 phosphorylation and mitochondrial translocation through the RhoA pathway, suggesting the involvement of S1P in mitochondrial fission (Brand et al., 2018). In human renal glomerular endothelial cells (HRGECs) reduction in Drp1 phosphorylation was achieved by blocking S1P via S1PR2 (Chen et al., 2019). Furthermore, manipulating the expression of S1PR1 disrupted the gene expression balance of mitochondrial fission and fusion genes (Bajwa et al., 2015). Therefore, these studies open a new perspective to explore S1P/S1PR signaling in mitochondrial dynamics. Another study revealed that blocking S1P using an SK inhibitor suppressed mitochondrial function and reduced mitochondrial fusion, evident from decreased expression of OPA1 and MFN1 (Hong et al., 2018). Moreover, as mentioned above, S1P regulates PGC1α, a key regulator of various mitochondrial proteins, including MFN2 (Figure 2). Hence, it can be speculated that S1P may cooperate with PGC1α to regulate mitochondrial fusion. The exact mechanism of how S1P controls mitochondrial dynamics remains unknown, but considering its involvement in multiple cellular pathways, it is plausible that S1P regulates the transcriptional and/or post-transcriptional expression of fission and fusion proteins. On the other hand, fission and fusion may be downstream, indirect effects of S1P on mitochondrial function including increased ROS expression, which may damage mitochondria and lead to fission. At any rate, further exploration is warranted in this area.

### Complex sphingolipids and mitochondria

While ceramide and S1P are extensively studied sphingolipids in the mitochondrial context, studies have also revealed involvement of complex sphingolipid species in mitochondrial biology. For example, ganglioside GD3, a glycosphingolipid with two sialic acid residues, has been shown to directly interact with mitochondria inducing mitochondrial permeability, cytochrome c release, and activation of apoptosis (Kristal and Brown, 1999; Scorrano et al., 1999; Garcia-Ruiz et al., 2000; Colell et al., 2001). Furthermore, studies utilizing immunoelectron and laser confocal microscopy in multiple cell lines including hepatocytes, human colon cells, and lymphoblasts have also demonstrated direct interaction and buildup of GD3 in mitochondria (Rippo et al., 2000; Colell et al., 2002; Garcia-Ruiz et al., 2002). Sphingomyelinase isoforms have also been found in the mitochondria in rodents and multiple cell lines whereas a novel mitochondria-associated neutral sphingomyelinase (MA-nSMase) has been discovered in murine model (Wu et al., 2010; Rajagopalan et al., 2015; Silvera et al., 2021). However, the specific impact of sphingomyelinase within the mitochondria remains to be elucidated. In addition to ceramide, sphingosine has also been shown to regulate mitochondrial cell death where exogenous sphingosine in Jurkat cells and MCF-7 induced mitochondrial cytochrome c release ultimately leading to cell death (Cuvillier et al., 2000; Cuvillier et al., 2001). Collectively, these studies establish a precedent for further exploration of complex sphingolipids in the context of mitochondrial interactions.

## Sphingolipids in mitochondria-based disease

### Sphingolipids and mitochondrial function in Parkinson’s disease

Parkinson’s disease (PD) is a neurodegenerative disorder characterized by motor and non-motor symptoms, primarily resulting from the progressive loss of dopaminergic neurons in the substantia nigra region of the brain. A defining pathological feature of PD is the presence of Lewy bodies which are caused by protein aggregates. Mitochondrial damage plays a pivotal role in the pathology of Lewy bodies, frequently occurring alongside lipid aggregates. This suggests a link between lipid homeostasis and mitochondrial damage in PD (Gai et al., 2000; Bras et al., 2008). Sphingolipid homeostasis, specifically, has been implicated in PD with studies showing genetic variants in Glucocerebrosidase (GCase), key enzyme in ceremide synthesis, to be the most common risk factor for PD (Sidransky et al., 2009). Furthermore, several studies have shown elevated levels of ceramide in various PD models. For instance, in a study using flies as a PD model, the downregulation of CPE, an analogue of SM in flies, and the upregulation of ceramides were observed. Additionally, suppressing ceramide levels either genetically or pharmacologically mitigated the phenotypic effects of PD (Lin et al., 2018). While this study did not demonstrate the specific mechanism or identify the ceramide species responsible for inducing mitochondrial defects in their model, a separate study observed similar results in both a Pink1-related PD model in flies and Pink1-deficient patient-derived fibroblasts where they showed that ceramide accumulation exacerbated the effects of Pink1 deficiency by reducing β-oxidation and inhibiting ceramide accumulation rescued the Pink1-related PD phenotype (Vos et al., 2021). On the contrary, conflicting results were seen when human PD postmortem brain sections showed reduction of sphingomyelin and ceramides (Abbott et al., 2014). The conflicting data arises from the fact that previous studies primarily focused on ceramide accumulation without investigating the specific ceramide species involved. In various Parkinson’s disease (PD) models, there may be a preference for certain ceramide species. For instance, human brain sections from PD patients exhibited an accumulation of short-chain acyl ceramides compared to long-chain ceramides (Abbott et al., 2014). This underscores the importance of identifying specific ceramide and CerS isoforms involved in cellular processes. Moreover, mitochondrial localization greatly determines ceramide function, emphasizing the need for further research to assess this aspect in different PD models.

### Sphingolipids and mitochondria in cancer

Sphingolipids, particularly ceramide and S1P, have garnered considerable attention in cancer research. Ceramides, known for their tumor-suppressing properties, have been found to induce cell cycle arrest, apoptosis, and lethal mitophagy, thereby exerting anti-tumor effects. Additionally, exposure of cells to chemotherapy or radiation has been linked to increased ceramide levels, which in turn have been associated with the inhibition of tumor growth and angiogenesis, further reinforcing their role as tumor suppressors (Senchenkov et al., 2001; Ogretmen, 2018). However, it is worth noting that ceramides can also contribute to cancer cell survival, drug resistance, and metastasis (Ferro et al., 2020; Panigrahi et al., 2020). S1P exhibits contrasting effects compared to ceramide as it promotes cell survival, proliferation, angiogenesis, and migration, making it a potent pro-tumorigenic sphingolipid (Furuya et al., 2011; Ogretmen, 2018; Li et al., 2022).

As highlighted earlier in this review, ceramides play crucial roles in regulating mitophagy, a process essential in cancer biology. Mitophagy can promote tumor suppression by eliminating dysfunctional mitochondria or function as lethal mitophagy, depriving cancer cells of an important energy supply. Notably, the most common genetic alteration observed in acute myeloid leukemia (AML) patients is in Fms-like tyrosine kinase 3 (FLT3), a receptor for tyrosine kinase. FLT signaling in AML suppresses CerS1 expression. Targeting this signaling pathway in both *in vitro* and *in vivo* models has been shown to induce CerS1/C-18 ceramide generation, leading to lethal mitophagy. This effect is facilitated through the Drp1-mediated translocation of CerS1 to the mitochondria, highlighting the intricate interplay between sphingolipids, mitophagy, and cancer progression in specific contexts (Dany et al., 2016).

Mitochondria, being negatively charged organelles, exhibit a preference for the accumulation of positively charged ceramides like pyridinium analogs, and an important consideration for ceramide-based chemotherapeutic approaches (such as treatment with positively charged ceramide analogs) is that cancer cells tend to have mitochondria with comparatively higher negative charge (Chen, 1988). The accumulation of positively charged ceramides induces membrane permeabilization, ultimately leading to cell death in various cancer cell lines (Novgorodov et al., 2005). In fact, synthetic positively charged ceramide (LCL29) has been utilized to induce mitochondrial-induced cell death in human colon carcinoma cells, demonstrating their potential as therapeutic agents (Dindo et al., 2006). Furthermore, combination therapies with positively charged ceramide analogs have proven successful in cancer drug therapies. Boppana et al. (2015) showed that LCL29 administration combined with phototherapy enhanced mitochondrial ceramide accumulation leading to mitochondrial apoptosis and overall cell death in human head and neck squamous carcinoma (HNSCC). Similarly, Senkal et al. utilized LCL29 alone or in combination with gemcitabine, a chemotherapeutic agent, and observed a reduction in HNSCC progression both *in vivo* and *in vitro* (Senkal et al., 2006). These examples highlight the potential chemotherapeutic use of synthetic ceramides.

The opposing effects of ceramide and S1P highlight the intricate balance between these sphingolipids in cancer biology. Ceramide acts as a tumor suppressor by regulating mitophagy and preventing the accumulation of dysfunctional mitochondria, which can contribute to tumorigenesis. On the other hand, S1P promotes cancer progression by promoting cell proliferation and migration independently of mitochondrial function. Understanding the interplay between these sphingolipids and their respective roles in cancer development can provide valuable insights for the development of targeted therapies aimed at modulating their levels and activities to combat cancer. Furthermore, understanding the intricate mechanisms of sphingolipids and their involvement in mitophagy provides valuable insights into cancer progression and offers potential therapeutic avenues for intervention.

### Sphingolipids and mitochondria in NAFLD

Non-alcoholic fatty liver disease (NAFLD) is the most common liver disorder and the major cause underlying the increasing rates of hepatocellular carcinoma in the United States, and potentially other areas (Fazel et al., 2016). Common manifestations of NAFLD include hepatic lipogenesis, lipid droplet formation, triglyceride accumulation, inflammation, hepatocyte damage, fibrosis, cirrhosis, and increased risk of hepatocellular carcinoma (HCC). Accumulation of sphingolipids (SLs) may serve as one of the driving factors of NAFLD progression. Interestingly, it has been shown that the quality, that is the carbon backbone length and SL species, rather than the quantity of SLs is more important (McClain et al., 2007). Mitochondrial dysfunction has emerged as a critical molecular event in the development of hepatic injury associated with high-fat diets (Nagiec et al., 1994; Hornemann et al., 2006). Studies have indicated that autophagy, particularly mitophagy, plays a significant role in the progression of NAFLD *in vivo* (Li et al., 2018; Yamada et al., 2018; Zhou et al., 2019). For instance, the overexpression of sirtuin 3 (Sirt3), a mitochondrial NAD-dependent deacetylase, has been shown to enhance binp3-mediated mitophagy, resulting in reduced hepatocyte injury (Li et al., 2018). Furthermore, Sirt3 has been found to regulate the synthesis of mitochondrial ceramides by deacetylating CerS (Novgorodov et al., 2016). Therefore, investigating the mechanisms, regulation, and roles of acetylation of CerS enzymes, which has not been broadly addressed, could provide valuable insights for the development of therapeutic interventions.

Ceramides play a role in impairing mitochondrial function and facilitating necrotic cell death in hepatocytes. Specifically, hepatocyte necrosis induced by ceramides is influenced by factors such as the length of the acyl chain, concentration, and duration of exposure. This form of cell death is accompanied by adenosine triphosphate (ATP) depletion and mitochondrial depolarization, each of which has been associated with aberrant ceramide production (Arora et al., 1997). Therefore, it is crucial to investigate the specific sphingolipid species, particularly ceramides, that contribute to mitochondrial dysfunction leading to NAFLD. While many studies have examined the overall profile of sphingolipids in various NAFLD models, the cellular localization of sphingolipids has often been overlooked. Additionally, thought the N-acyl chain of ceramides has received much attention, recent advancements in our understanding of sphingoid base biosynthesis through SPT have provided new routes for studies, some of which have observed upregulation of the SPTLC3 subunit of serine palmitoyltransferase (SPT) in NAFLD and hepatocellular carcinoma (HCC) models (Yoshimine et al., 2015; Teng et al., 2019). However, detailed analyses of these non-canonical sphingolipids derived from SPTLC3, their downstream incorporation into ceramides, and their potential mechanistic contributions to NAFLD have not been conducted, which could further expand the possibilities for the development of therapeutic approaches.

## Conclusion and future direction

Sphingolipids are a broad and dynamic class of cellular lipid, and studies including those discussed here have established their functions in mitochondria, implicating them as potential players in various mitochondria-related pathologies and diseases (Hernandez-Corbacho et al., 2017; Czubowicz et al., 2019; Parveen et al., 2019; Roszczyc-Owsiejczuk and Zabielski, 2021). These important studies lay a solid foundation for further research to better understand the specific functions of ceramide synthases and complex sphingolipids derived from distinct N-acyl chain ceramides. Additionally, nearly all of these previous research efforts have focused on canonical sphingolipids, specifically ceramide and S1P, with an 18-carbon backbone, in relation to mitochondrial function. While these studies have provided valuable insights into the impact of these sphingolipids on various mitochondrial processes, the exploration of non-canonical sphingolipid species that vary in carbon chain length remains in its initial stages. Non-canonical sphingolipids encompass an emerging yet diverse array of sphingolipid species that differ in the sphingoid base including in carbon backbone length and configuration (i.e., methylation) and saturation (e.g. sphingadienes). To advance our understanding of sphingolipid-mediated mitochondrial processes, it is crucial to investigate the involvement of these alternative sphingolipids.

An additional consideration for future studies is the topology and specific cellular compartmentation of sphingolipid metabolism. Many studies have used and continue to use exogenously added sphingolipids including synthetic short- and medium-chain ceramides as well as sphingosine-1-phosphate. While these studies undoubtedly provide valuable information, the state of the field now encompasses intricacies in synthesis, intracellular transport, and compartment-specific metabolism that will undoubtedly be required to fully understand any sphingolipid-dependent mechanism studies. As discussed earlier in this review, genetic modifications and/or the pharmacological inhibition of specific sphingolipids can significantly contribute to this endeavor. Furthermore, a comprehensive understanding requires investigating the mitochondrial sphingolipid profile in multiple cell types and disease models, considering the interplay between different sphingolipids, receptors, and downstream signaling pathways. In summary, abundant evidence strongly supports continued investment in sphingolipids within the mitochondrial context. However, increased focus on nuances of biosynthesis, specific species (with respect not only to ceramide N-acyl chains but also to non-canonical sphingoid bases), dd intracellular transport, and compartmentalization, will provide a deeper understanding of their functional significance. This will be required to improve understanding of mechanisms, identify therapeutic targets, and develop disease interventions.
